# Global burden of stroke attributable to high systolic blood pressure in 204 countries and territories, 1990–2019

**DOI:** 10.3389/fcvm.2024.1339910

**Published:** 2024-04-26

**Authors:** Junxiao Li, Qiongqiong Zhong, Shixiang Yuan, Feng Zhu

**Affiliations:** ^1^ Central Laboratory, Guangzhou Twelfth People's Hospital, Guangzhou, China; ^2^ Departments of Public Health and Preventive Medicine, Jinan University, Guangzhou, China; ^3^ Department of Neurosurgery, Guangzhou Twelfth People’s Hospital, Guangzhou, China

**Keywords:** high systolic blood pressure, stroke, disability-adjusted life years, mortality, age-standardized rate

## Abstract

**Background:**

High systolic blood pressure (HSBP) is severely related to stroke, although the global burden of stroke associated with HSBP needs to be understood.

**Materials and methods:**

Data derived from the Global Burden of Disease, Injuries, and Risk Factors Study were used to analyze deaths, disability-adjusted life years (DALYs), age-standardized rates of mortality (ASMR), age-standardized rates of DALY (ASDR), and estimated annual percentage change (EAPC).

**Results:**

Globally, 52.57% of deaths and 55.54% of DALYs from stroke were attributable to HSBP in 2019, with higher levels in men; the ASMRs and ASDRs in 1990–2019 experienced a decline of 34.89% and 31.71%, respectively, with the highest ASMR- and ASDR-related EAPCs in women. The middle socio-demographic index (SDI) regions showed the most numbers of deaths and DALYs in 2019 and 1990, with a decline in ASMR and ASDR; East Asia shared over 33% of global deaths and DALYs; Central Asia shared the highest ASMR and ASDR; high-income Asia Pacific experienced the highest decline in the ASMR- and ASDR-related EAPCs. Central and Southeast Asia had the highest percentages for deaths and DALYs, respectively, with more ASMR in high-middle SDI; the SDI and human development index were negatively associated with ASMR/ASDR and ASMR/ASDR-related EAPCs in 2019.

**Conclusion:**

Global deaths and DALYs of stroke attributable to HSBP but none of their age-standardized rates have been on the rise over the past three decades; its disease burden focused especially on men aged 70 years and older in East, Central, and Southeast Asia, and the middle to high SDI regions.

## Introduction

Stroke is a severe public health problem worldwide (1). Recent data showed that deaths from stroke experienced a rise of 16.6% between 2007 and 2017, and its corresponding years of life lost (YLLs) ranked third in the Global Burden of Diseases, Injuries, and Risk Factors Study (GBD) cause hierarchy, with rises of 12.9% between 1990 and 2007 and 12.1% between 2007 and 2017 (2, 3); in 2019, stroke became the top-ranked cause of disability-adjusted life years (DALYs) in populations aged over 50 years (4). Globally, 1.12% of the gross domestic product (GDP) was taken up by the aggregate costs of stroke in 2017 (5). High systolic blood pressure (HSBP) is a commonly severe threat to human health; exposure to HSBP experienced a minor decline (−1.37%) but a rise in deaths (22.8%) and DALYs (20.0%) due to HSBP between 1990 and 2017 (3). HSBP, a severe risk of most cardiovascular diseases, has been influencing health in populations aged 25 years and older because it was the first and second mortality risk in women and men, respectively, and the first and second leading risk factor for DALYs in all ages and those aged over 50 years and populations aged 25–49 years in 2019, respectively (4); therefore, blood pressure control has been a pivotal preventive intervention strategy (6). Accelerating evidence demonstrates that HSBP is the foremost risk factor for stroke, with a rating of 1/19 for all strokes (contributing to 79.6 million DALYs or 55.5% of overall stroke DALYs) (7) and a rise of 52.6% in deaths between 2007 and 2017 worldwide (3); therefore, stroke attributable to HSBP should progressively be paid more attention, and anti-HSBP tactics, including targeting and controlling SBP level, have been better-known approaches in the management of the global HSBP-related disease burden. However, the epidemiological pattern of stroke attributable to HSBP remains unclear; therefore, elucidating the epidemiological characteristics of stroke related to HSBP to develop precise preventive strategies is particularly important.

The GBD is available by combining new datasets, enhancing method performance and standardization, and answering scientific outbreaks. Human diseases can cause various premature deaths and disabilities; the corresponding progress and adverse patterns need to be assessed. Here, we used data from the GBD 2019 to estimate the trends of all strokes (ischemic and hemorrhagic) attributable to HSBP in deaths, DALYs, their age-standardized rates (ASRs), and their age, sex, global, regional, and national levels.

## Methods

### Data source and study sample

Study data on the disease burden of stroke attributable to HSBP between 1990 and 2019, including annual cases of deaths/DALYs and their age-standardized rates of mortality (ASMR) and DALY (ASDR) by sex, age, and location, were extracted from the GBD 2019 [a publicly available resource (http://ghdx.healthdata.org/gbd-results-tool) recorded 369 diseases and injuries in 21 regions and 204 countries and territories]. This study complied with the Guidelines for Accurate and Transparent Health Estimates Reporting (GATHER) guidelines.

### HSBP definition and stroke identification

A SBP of at least 110–115 mmHg was defined as HSBP according to a theoretical minimum exposure aimed at capturing the maximum-caused burden, which was related to a higher risk of cardiovascular diseases (particularly stroke). The detailed data selection and input have been described previously (6). Each cause and related state of stroke, covering the years 1990–2019, was defined according to the World Health Organization (WHO) criteria and the International Statistical Classification of Diseases (ICD, the 10th Related Health Problems) (8), and the Cause List Mapped to the ICD codes of I60–I62, I62.9–I64, I64.1, I65–I69.998, Z82.3, and G45–46.8 (9, 10). Stroke attributable to HSBP was defined as a stroke combining exposure to the risk-related HSBP (11).

### Disease burden measurement and statistical analyses

Disease burden was quantified by the numbers of deaths, DALYs, ASMR, and ASDR, with 95% uncertainty intervals (UI). The socio-demographic index (SDI), a composite index of per capita income, educational attainment, and total fertility rate of all areas, can be derived from the Global Health Data Exchange (GHDx, http://ghdx.healthdata.org), wherein the socio-demographic development status in different countries or regions are identified, and the geometric mean on a scale of 0–1 is calculated, by which five groups are low, low-middle, middle, middle-high, and high countries (12). Among 204 countries and territories, 5 SDIs and 21 GBD regions were available to assess the disease burdens by location, according to epidemiological and geographical conditions. More details on the SDI calculation have been described previously (4, 13), wherein ages were categorized into 15 groups from 25 to 94 years and older than 95 years.

The GBD 2019 comparative risk assessment approach was used to estimate population fractions. A no-weighted mean of the GBD year's age-related proportional distributions for national locations, with more than 5 million populations in the GBD year to update the global population age standard (4), was used to calculate ASR. In brief, several parameters were summed up to calculate the following: the age-standardized rate products (*a_i_*, wherein *i* is the *i*th age class), a number (or the weight) of persons (*w_i_*) in the same age subgroup I (a standard population reference), and a dividend of the standard population weights (14, 15); in the formula of $\mathrm{ASR}=\sum_{i=1}^{A}a_{i}w_{i}/\sum_{i=1}^{A}w_{i}\times 100,000$, ASMR (per 100,000 person-years) and ASDR (per 100,000 people) estimates are presented according to WHO's standard population reference (16), with 95% UIs for every metric.

The estimated annual percentage change (EAPC) can quantify the secular trend of ASRs according to a previously well-established formula (17): ln(*y*) = *α* + *βx* + *ε*, wherein *y* = ln(ASR), *x* = calendar year, and *ε* = the error term. Its 95% confidence interval (CI) was from a linear regression model; the absolute EAPC value represents the rate changes over time; the smoothing splines model fitted the association between EAPC and SDI; ASMR or ASDR was recognized to be on the rise if the EAPC estimation and 95% CI > 0, a decreasing trend if the EAPC estimation and 95% CI < 0, and stable if 95% CI = 0 (4). The Pearson correlation analysis was used to measure the degree of correlation between two variables; for the influential factors for EAPC, the associations between EAPC and SDI, ASR, and human development index (HDI) at the national level were assessed (18). Based on the social development and health outcomes in 2019 and the ASR in 1990, the HDI in 2019 was referred to as a national healthy condition. A hierarchical cluster analysis was conducted in four categories of countries and territories ((1) minor increase; (2) remain stable; (3) minor decrease; (4) significant decrease) in accordance with the temporal trends in ASMR and ASDR by a join point regression model (version 4.9.1.0), wherein the *Z* Test was used, with a two-sided *P* value of 0.05 as a significant level. A traditional linear regression was used when H0 (a segmentation point as 0) was first assumed, wherein H1 was a segmentation with at least 1 point; once H0 was rejected, the significance of the segment points of 1 − *n* was estimated. According to the estimate of annual percentage change (APC) (19), the time series and the ASRs were set up and taken as independent and dependent variables, respectively. R (version 4.2.1; R Core Team, Vienna, Austria) was used in the visualization of indicators and linear correlations and coefficients between the ASRs and SDI; *P *< 0.05 was defined as significance; draw maps were conducted in a visualization tool [GBD Compare, IHME Viz Hub (healthdata.org)].

## Results

### The global trends of stroke attributable to HSBP

**Figure 1:**
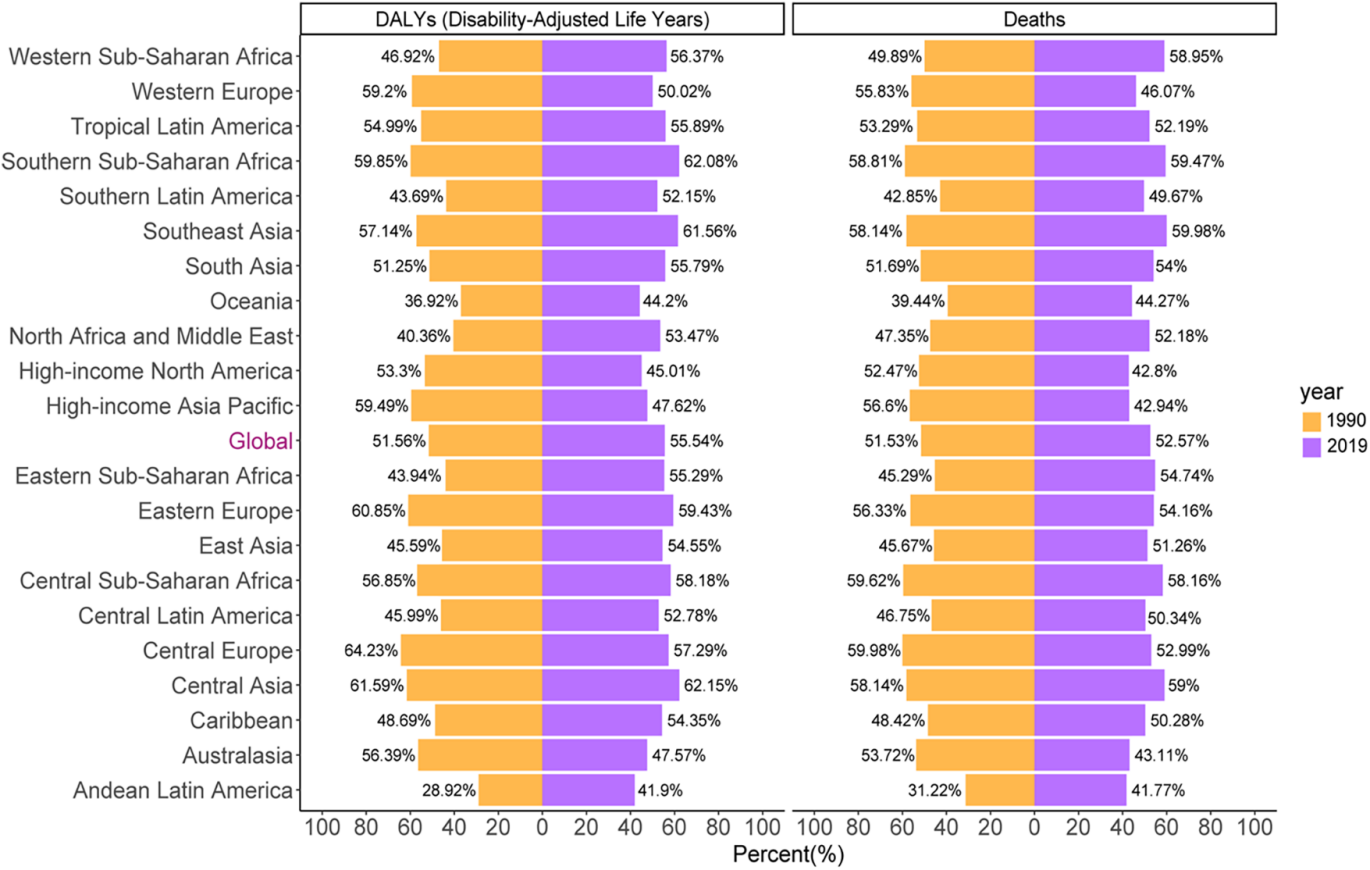
The proportion of deaths and DALYs from stroke attributable to HSBP worldwide and in 21 GBD regions in the years 1990 and 2019.

**Figure 2:**
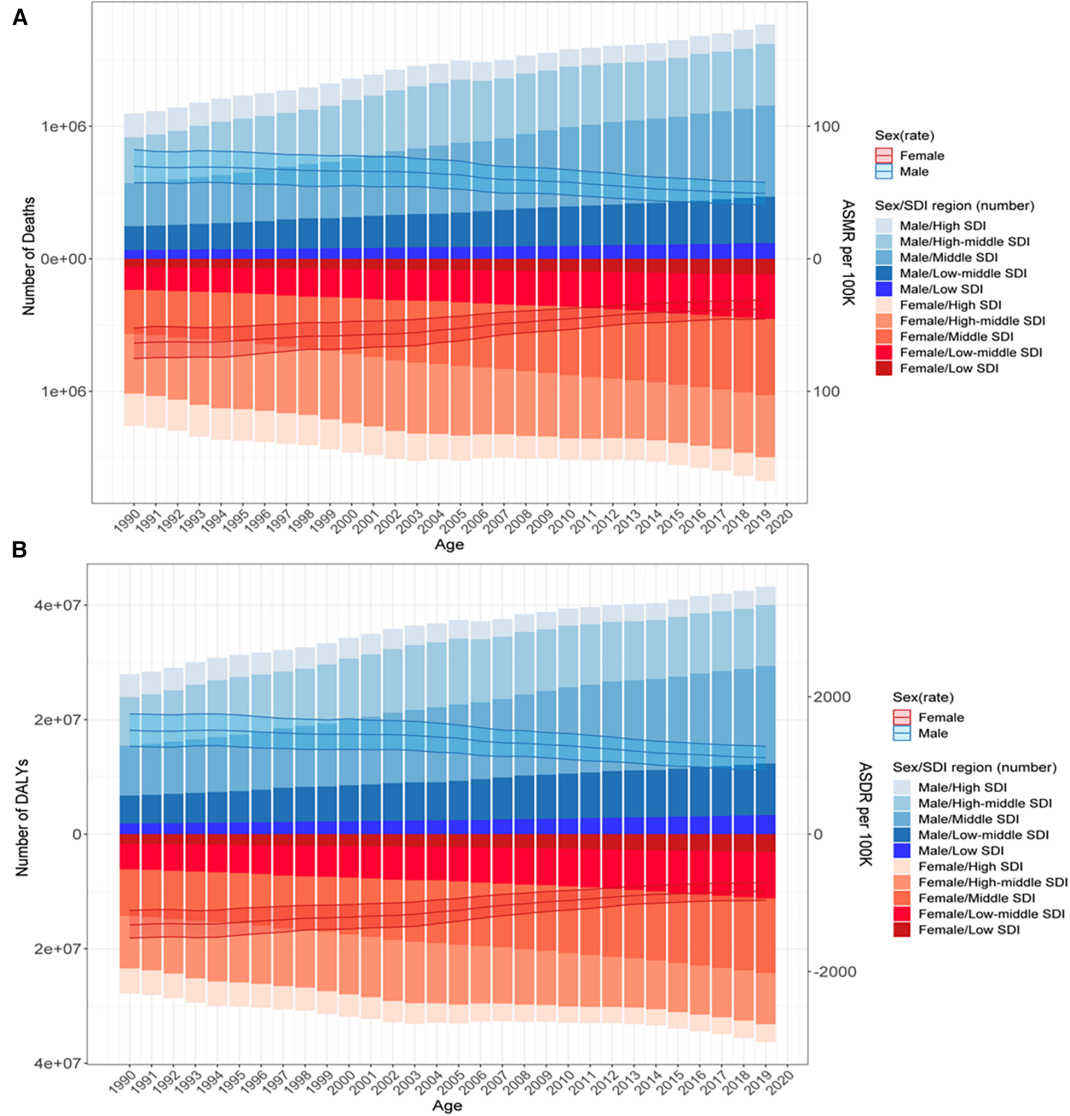
Deaths (**A**) and DALYs (**B**) of stroke attributable to HSBP in 1990–2019 by sex and SDI region. The bar was the number of deaths and DALYs by SDI level. The line with 95% UI represents ASMR and ASDR at the global level.

Globally, 52.57% of deaths and 55.54% of DALYs from stroke were due to HSBP, with approximately 3.44 million in 2019 and 2.36 million in 1990 for deaths, and 79.6 million in 2019 and 58.8 million in 1990 for DALYs; furthermore, men bore more deaths and higher DALYs (Figure 1). The corresponding ASMRs (66.96 per 100,000, 95% UI: 55.11–78.88 in 1990 vs. 43.6 per 100,000, 95% UI: 36.19–51.06 in 2019) and ASDRs (1,419.59 per 100,000, 95% UI: 1,197.05–1,629.35 in 1990 vs. 969.4 per 100,000, 95% UI: 823.77–1,110.2 in 2019) experienced a decline by 34.89% and 31.71%, respectively, with the highest levels of the ASMR-related EAPC (−1.99, 95% CI: −2.12 to −1.85) and ASDR-related EAPC (−1.81, 95% CI: −1.93 to −1.69) in women (Table 1 and Figure 2).

**Table 1:** Global burden in 1990 and 2019 and the temporal trends from 1990 to 2019 in stroke attributable to high systolic blood pressure.

Characteristics	1990				2019				EAPC (1990–2019)	
	Death cases	ASMR per 100k	DALYs	ASDR per 100k	Death cases	ASMR per 100k	DALYs	ASDR per 100 k	ASMR	ASDR
	No. ×10^5^ (95% UI)	No. (95% UI)	No. ×10^5^ (95% UI)	No. (95% UI)	No. ×10^5^ (95% UI)	No. (95% UI)	No. ×10^5^ (95% UI)	No. (95% UI)	No. (95% CI)	No. (95% CI)
Global	23.57 (19.72 to 27.45)	66.96 (55.11 to 78.88)	557.99 (476.04 to 640.15)	1,419.59 (1,197.05 to 1,629.35)	34.44 (28.66 to 40.09)	43.6 (36.19 to 51.06)	795.57 (676.54 to 907.55)	969.4 (823.77 to 1,110.2)	−1.64 (−1.76 to −1.52)	-1.46 (−1.57 to −1.35)
Sex										
Female	12.6 (10.39 to 14.69)	63.67 (52.31 to 75.28)	278.44 (234.92 to 319.05)	1,322.01 (1,109.63 to 1,512.21)	16.76 (13.57 to 19.98)	38.21 (30.96 to 45.51)	363.01 (307.09 to 420.01)	832.43 (704.29 to 962.10)	−1.99 (−2.12 to −1.85)	−1.81 (−1.93 to −1.69)
Male	10.97 (9.16 to 12.78)	69.78 (57.48 to 82.35)	279.55 (234.80 to 322.83)	1,513.96 (1,280.54 to 1,750.02)	17.68 (14.66 to 20.49)	49.44 (40.57 to 57.62)	432.56 (362.30 to 495.86)	1,113.34 (929.33 to 1,278.51)	−1.28 (−1.39 to −1.16)	−1.14 (−1.25 to −1.02)
SDI region										
High SDI	4.21 (3.43 to 4.99)	40.12 (32.78 to 47.63)	83.94 (72.14 to 95.19)	808.83 (698.12 to 917.31)	3.25 (2.5 to 4.09)	15.15 (12.02 to 18.49)	63.03 (52.47 to 74.35)	345.13 (290.83 to 402.95)	−3.86 (−4.08 to −3.65)	−3.38 (−3.58 to −3.18)
High-middle SDI	7.97 (6.69 to 9.18)	84.54 (70.08 to 99.63)	176.79 (151.75 to 201.12)	1,689.54 (1,443.55 to 1,925.96)	9.32 (7.56 to 11.03)	46.81 (38.06 to 55.58)	196.37 (167.06 to 225.45)	972.73 (827.47 to 1,117.39)	−2.42 (−2.68 to −2.17)	−2.28 (−2.54 to −2.02)
Middle SDI	6.61 (5.36 to 7.81)	76.96 (61.7 to 92.64)	168.37 (138.96 to 198.53)	1,641.9 (1,352.85 to 1,933.12)	12.64 (10.51 to 14.7)	57.07 (47.02 to 67.25)	300.62 (254.31 to 345.66)	1,215.12 (1,028.78 to 1,396.49)	−0.94 (−1.11 to −0.76)	−0.96 (−1.1 to −0.83)
Low-middle SDI	3.5 (2.87 to 4.19)	68.51 (54.99 to 83.06)	93.03 (77.56 to 109.55)	1,519.02 (1,263.09 to 1,793.28)	6.83 (5.74 to 7.86)	55.24 (45.85 to 64.45)	170.21 (142.84 to 195.24)	1,227.78 (1,035.14 to 1,403.72)	−0.79 (−0.9 to −0.68)	−0.77 (−0.86 to −0.68)
Low SDI	1.28 (1 to 1.58)	62.45 (47.4 to 78.07)	35.6 (28.6 to 43.13)	1,439.11 (1,143.99 to 1,750.68)	2.38 (1.91 to 2.86)	52.11 (41.91 to 63.39)	64.89 (52.96 to 77.21)	1,191.78 (969.1 to 1,418.07)	−0.63 (−0.71 to −0.54)	−0.66 (−0.73 to −0.6)
GBD region										
Andean Latin America	0.04 (0.03 to 0.06)	23.04 (17.45 to 28.88)	1.15 (0.88 to 1.42)	532.64 (409.01 to 653.26)	0.08 (0.06 to 0.11)	15.53 (11.5 to 20.82)	1.97 (1.5 to 2.54)	345.76 (263.79 to 445.22)	−1.15 (−1.49 to −0.8)	−1.37 (−1.71 to −1.03)
Australasia	0.07 (0.06 to 0.09)	33.36 (26.54 to 41.03)	1.39 (1.18 to 1.61)	600.33 (508.07 to 695)	0.07 (0.05 to 0.09)	11.93 (8.93 to 15.32)	1.1 (0.89 to 1.33)	222.23 (182.39 to 265.99)	−4.06 (−4.28 to −3.84)	−3.85 (−4.09 to −3.61)
Caribbean	0.12 (0.1 to 0.14)	47.17 (38.36 to 56.61)	2.87 (2.38 to 3.36)	1,087.28 (899.3 to 1,273.18)	0.2 (0.16 to 0.24)	37.9 (29.97 to 47.11)	4.62 (3.63 to 5.63)	894.88 (702.95 to 1,088.46)	−0.71 (−0.82 to −0.6)	−0.62 (−0.76 to −0.48)
Central Asia	0.38 (0.33 to 0.43)	89.22 (76.2 to 101.38)	9.45 (8.29 to 10.46)	2,009.59 (1,753.72 to 2,236.53)	0.54 (0.46 to 0.63)	89.6 (74.61 to 104.23)	13.7 (11.59 to 15.72)	1,866.16 (1,575.09 to 2,145.87)	−0.3 (−0.67 to 0.08)	−0.59 (−0.93 to −0.24)
Central Europe	1.33 (1.13 to 1.52)	96.92 (81.7 to 111.71)	29.17 (25.49 to 32.33)	2,006.34 (1,754.56 to 2,237.09)	1.14 (0.89 to 1.4)	50.8 (40.07 to 62.67)	21.13 (17.23 to 25.21)	1,000.31 (821.19 to 1,189.02)	−2.67 (−2.89 to −2.44)	−2.87 (−3.09 to −2.66)
Central Latin America	0.23 (0.19 to 0.27)	30.75 (25.33 to 36.11)	5.83 (4.94 to 6.68)	672.73 (569.77 to 770.3)	0.44 (0.35 to 0.55)	19.47 (15.27 to 24.28)	10.25 (8.3 to 12.45)	430.21 (350.36 to 522.27)	−1.86 (−1.98 to −1.74)	−1.82 (−1.95 to −1.68)
Central sub-Saharan Africa	0.16 (0.12 to 0.21)	83.32 (61.82 to 106.33)	4.57 (3.53 to 5.76)	1,919.38 (1,479.46 to 2,425.75)	0.29 (0.21 to 0.37)	65.48 (47.92 to 86.83)	7.94 (5.9 to 10.14)	1,445.41 (1,076.34 to 1,858.93)	−0.89 (−1.03 to −0.76)	−1.05 (−1.17 to −0.93)
East Asia	6.49 (5.04 to 8.1)	92.14 (69.55 to 115.33)	158.5 (122.43 to 196.17)	1,860.34 (1,452.08 to 2,289.12)	11.58 (9.16 to 14.08)	62.34 (48.82 to 76.28)	259.59 (210.4 to 310)	1,271.49 (1,028.97 to 1,514.34)	−1.3 (−1.54 to −1.07)	−1.3 (−1.49 to −1.1)
Eastern Europe	2.75 (2.28 to 3.17)	107.31 (87.6 to 126.73)	58.86 (50.55 to 66.17)	2,139.14 (1,831.29 to 2,421.89)	2.47 (1.98 to 2.99)	71.5 (58.03 to 86.58)	50.29 (42.11 to 58.46)	1,498.29 (1,259.64 to 1,734.63)	−2.19 (−2.68 to −1.71)	−1.99 (−2.49 to −1.49)
Eastern sub-Saharan Africa	0.43 (0.33 to 0.52)	63.58 (47.81 to 78.86)	12.04 (9.47 to 14.74)	1,509.64 (1,176.47 to 1,851.91)	0.85 (0.65 to 1.04)	60.05 (45.8 to 74.36)	23.15 (18.16 to 28.03)	1,350.33 (1,056.24 to 1,637.27)	−0.18 (−0.22 to −0.14)	−0.4 (−0.44 to −0.36)
High-income Asia Pacific	1 (0.84 to 1.16)	55.69 (45.99 to 65.9)	22.15 (19.14 to 24.96)	1,123.67 (968.58 to 1,272.32)	0.78 (0.58 to 1.01)	14.33 (11.1 to 17.73)	15.45 (12.54 to 18.55)	364.46 (301.58 to 429.54)	−5.17 (−5.38 to −4.96)	−4.34 (−4.53 to −4.16)
High-income North America	0.88 (0.72 to 1.06)	24.09 (19.58 to 28.79)	18.26 (15.5 to 21.22)	518.09 (440.18 to 600.82)	0.89 (0.68 to 1.12)	13.05 (10.23 to 16.15)	18.7 (15.11 to 22.21)	308.83 (252.63 to 366.22)	−2.71 (−2.99 to −2.42)	−2.23 (−2.49 to −1.97)
North Africa and Middle East	0.84 (0.68 to 1.01)	58.73 (46.65 to 71.67)	22.37 (18.5 to 26.45)	1,285.31 (1 to 054.81 to 1,530.54)	1.63 (1.33 to 1.95)	43.96 (35.07 to 53.46)	42.5 (35.17 to 50.38)	965.01 (800.6 to 1,143.24)	−0.98 (−1.03 to −0.92)	−0.99 (−1.04 to −0.93)
Oceania	0.01 (0.01 to 0.02)	57.33 (40.85 to 76.87)	0.45 (0.33 to 0.61)	1,411.05 (1,035.16 to 1,852.77)	0.04 (0.03 to 0.05)	56.17 (40.14 to 75.21)	1.18 (0.85 to 1.62)	1,467.75 (1,076.86 to 1,959.87)	−0.11 (−0.19 to −0.02)	0.16 (0.01 to 0.3)
South Asia	2.71 (2.2 to 3.25)	58.01 (46.34 to 71.4)	72.58 (59.84 to 86.45)	1,258.76 (1,034.65 to 1,505.65)	5.29 (4.28 to 6.23)	41.5 (33.36 to 49.31)	134.59 (110.36 to 156.8)	936.02 (768 to 1,091.23)	−1.31 (−1.46 to −1.15)	−1.1 (−1.21 to −0.99)
Southeast Asia	2.05 (1.72 to 2.43)	93.02 (76.02 to 111.8)	55.36 (46.89 to 64.49)	2,114.83 (1,785.44 to 2,465.14)	4.39 (3.72 to 5.05)	81.59 (68.07 to 94.51)	111.96 (94.74 to 128.16)	1,817.25 (1,555.88 to 2,072.88)	−0.2 (−0.36 to −0.05)	−0.29 (−0.42 to −0.16)
Southern Latin America	0.18 (0.15 to 0.22)	41.89 (33.92 to 50.7)	4.24 (3.5 to 4.96)	924.84 (762.59 to 1,082.22)	0.2 (0.16 to 0.24)	23.47 (19.14 to 28.01)	4.12 (3.47 to 4.74)	501.56 (422.89 to 576)	−2.03 (−2.11 to −1.95)	−2.18 (−2.25 to −2.11)
Southern sub-Saharan Africa	0.13 (0.11 to 0.15)	51.79 (42.22 to 62.05)	3.48 (2.93 to 4.03)	1,204.58 (1,005.75 to 1,400.46)	0.24 (0.2 to 0.28)	49.96 (41.5 to 59.02)	5.87 (5.03 to 6.69)	1,049.16 (896.38 to 1,202.48)	−0.04 (−0.55 to 0.47)	−0.41 (−0.89 to 0.07)
Tropical Latin America	0.57 (0.49 to 0.65)	69.39 (58.08 to 80.77)	15.48 (13.18 to 17.52)	1,603.05 (1,370.51 to 1,812.87)	0.7 (0.59 to 0.82)	29.91 (24.81 to 35.04)	16.4 (14.02 to 18.56)	671.23 (573.11 to 761.98)	−3.02 (−3.09 to −2.94)	−3.17 (−3.24 to −3.09)
Western Europe	2.71 (2.19 to 3.26)	45.53 (36.78 to 54.9)	47.5 (40.5 to 54.05)	817.05 (703.55 to 926.84)	1.71 (1.28 to 2.18)	15.43 (12.06 to 19.16)	26.41 (21.66 to 31.32)	286.83 (242.88 to 332.05)	−4.21 (−4.43 to −3.98)	−4.03 (−4.24 to −3.82)
Western sub-Saharan Africa	0.47 (0.36 to 0.6)	62.68 (47.79 to 80.22)	12.27 (9.61 to 15.35)	1,374.13 (1,073.53 to 1,732.11)	0.93 (0.75 to 1.11)	58.65 (47.3 to 70.79)	24.63 (19.88 to 29.82)	1,263.43 (1,026.1 to 1,507.35)	−0.08 (−0.2 to 0.04)	−0.14 (−0.25 to −0.03)

No., number.

### The regional trends of stroke attributable to HSBP

Significant geographical variations in the GBD region-related deaths and DALYs showed disparities over the past three decades. Middle SDI regions had the most number of deaths (12.64 × 10^5^, 95% UI: 10.51–14.7 in 2019 vs. 6.61 × 10^5^, 95% UI: 5.36–7.81 in 1990) and DALYs (300.62 × 10^5^, 95% UI: 254.31–345.66 in 2019 vs. 168.37 × 10^5^, 95% UI: 138.96–198.53 in 1990), with both accounting levels over 35% worldwide; middle and low-middle SDI regions had the highest levels of ASMR (57.07 per 100,000, 95% UI: 47.02–67.25) and ASDR (1,215.12 per 100,000, 95% UI: 1,028.78–1,396.49). Notably, the ASMR and ASDR between 1990 and 2019 experienced a decline across the five SDI regions, with the largest decline in the ASMR-related EAPC (−3.86, 95% CI: −4.08 to −3.65) and ASDR-related EAPC (−3.38, 95% CI: −3.58 to −3.18) in high SDI regions (Table 1).

Across the GBD regions in 2019, East Asia had over 33% of global deaths (11.58 × 10^5^, 95% UI: 9.16–14.08) and DALYs (259.59 × 10^5^, 95% UI: 210.4–310); Central Asia had the highest levels of ASMR (89.6 per 100,000, 95% UI: 74.61–104.23) and ASDR (1,866.16 per 100,000, 95% UI: 1,575.09–2,145.87). The ASMR-related EAPCs between 1990 and 2019 were <0, with the largest decline in high-income Asia Pacific (EAPC = −5.17, 95% CI: −5.38 to −4.96); the ASDR-related EAPCs in most of the GBD regions between 1990 and 2019 were <0, with only one level >0 in Oceania (EAPC = 0.16, 95% CI: 0.01–0.3) and the largest decline in high-income Asia Pacific (EAPC = −4.34, 95% CI: −4.53 to −4.16) (Table 1).

The percentages of deaths and DALYs in 1990 showed an approximately twofold difference, with their highest levels in Eastern and Central Europe, Central Asia, and southern sub-Saharan Africa but their lowest levels in Andean Latin America, Oceania, and southern Latin America. These data in 2019 accounted for over 40% of all GBD regions, with the highest percentages of deaths in Central Asia and DALYs in Southeast Asia (Figure 1).

### The countries and territories’ trends of stroke attributable to HSBP

**Figure 3:**
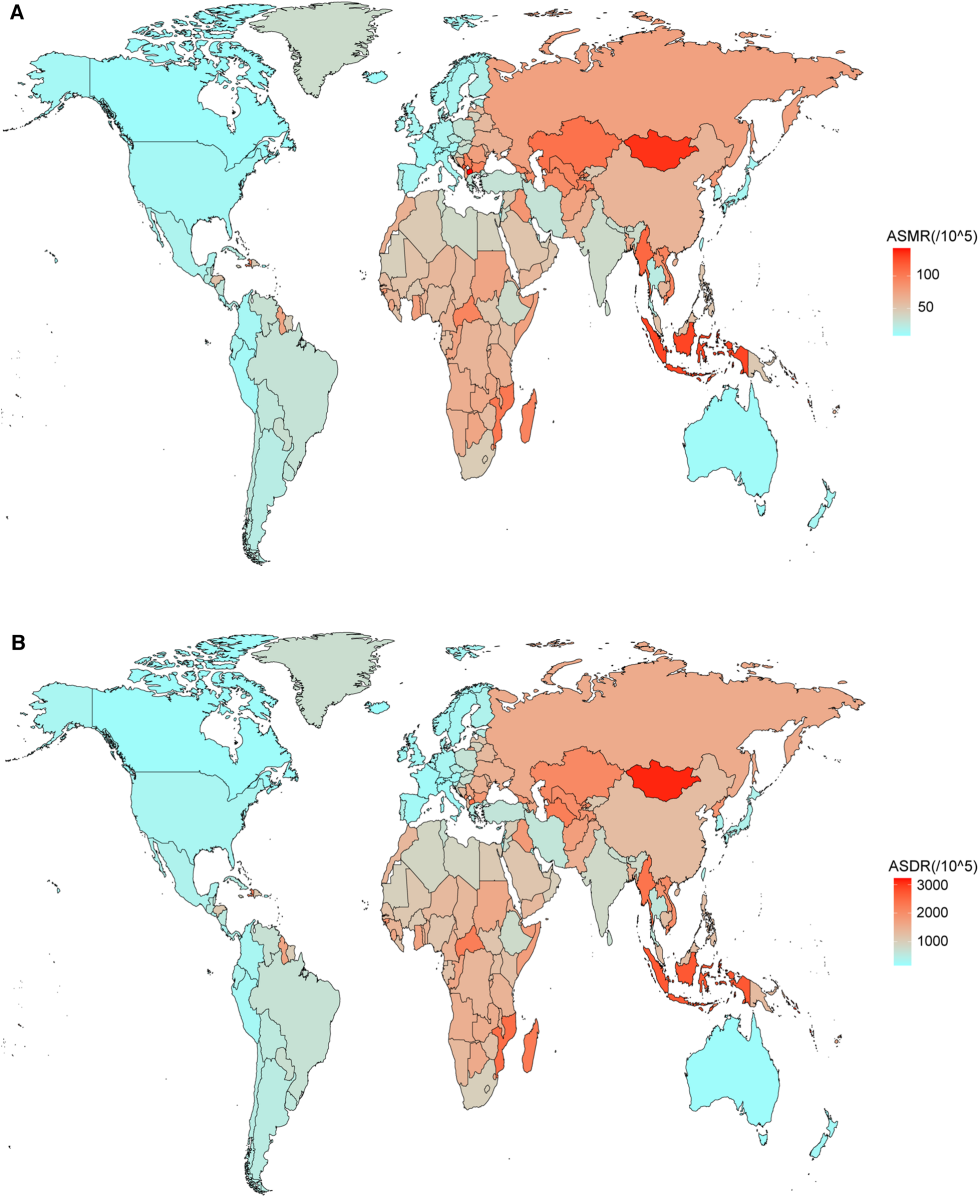

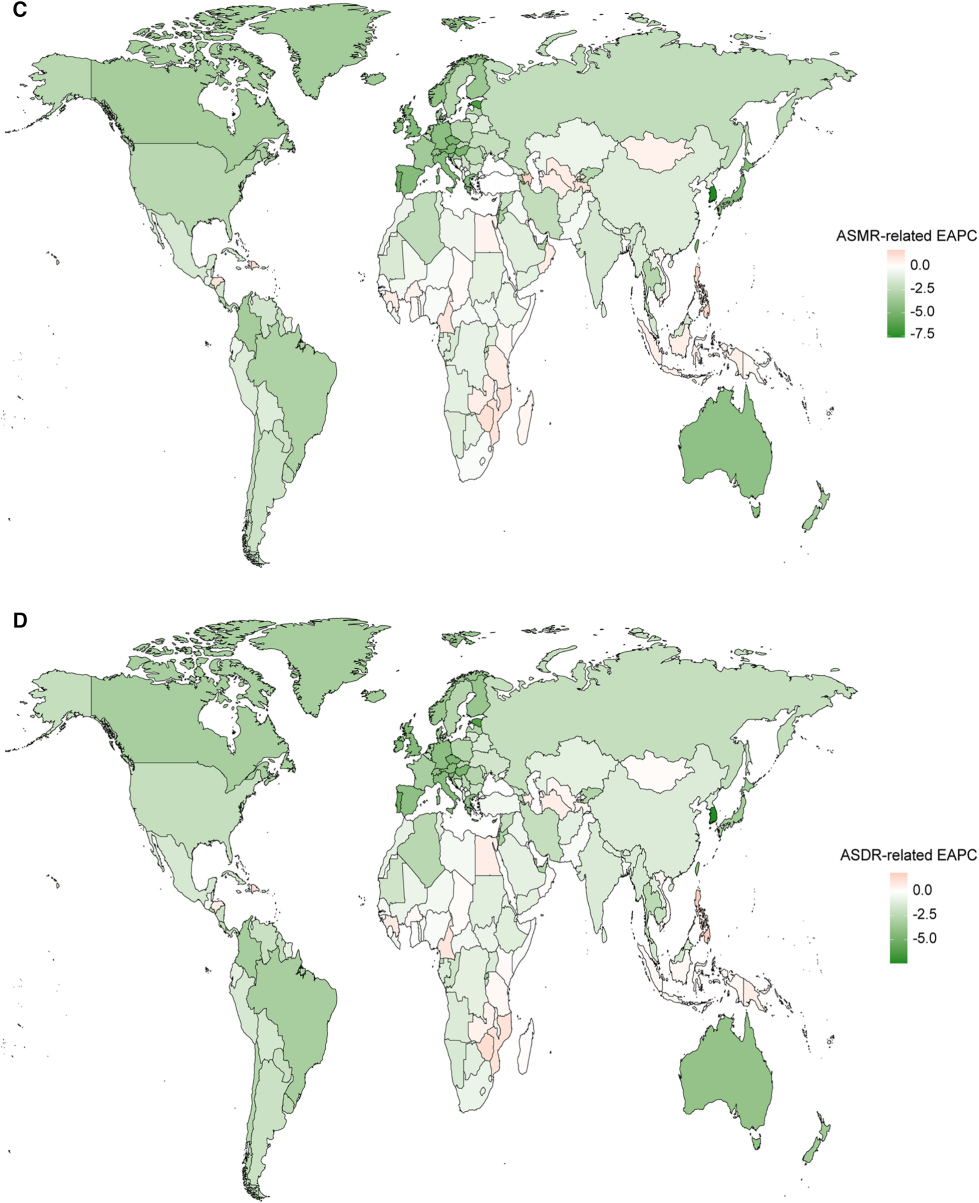
Spatial distribution of ASMR (**A**), ASDR (**B**), ASMR-related EAPC (**C**), and ASDR-related EAPC (**D**) of stroke attributable to HSBP in 2019.

China ranked the highest in the number of deaths (1,126,038, 95% UI: 889,923–1,370,837) and DALYs (25,176,256, 95% UI: 20,271,928–30,148,436) (Table 2). North Macedonia (142.76, 95% UI: 105.37–187.27), Mongolia (134.57, 95% UI: 101.96–174.56), and Indonesia (128.32, 95% UI: 105.53–149.57) shared the top three ASMRs; Nauru (3,223.45, 95% UI: 2,461.86–4,130.88), Mongolia (3,111.56, 95% UI: 2,378.13–4,060.13), and Vanuatu (3,056.07, 95% UI: 2,281.04–4,064.91) shared the top three ASDRs in 2019 (Figures 3A,B and Table 2). Azerbaijan had the highest rise in ASMR (ASMR-related EAPC = 1.79, 95% CI: 1.39–2.19) and the Philippines had the highest rise in ASDR (ASDR-related EAPC = 1.94, 95% CI: 1.34–2.55) (Figures 3C,D and Table 2).

**Table 2:** Top 10 countries or territories with the most number of deaths and DALY, the highest ASMR, and the highest EAPC in stroke attributable to high systolic blood pressure in 2,019.

Countries or territories	Number of deaths [No. (95% UI)]	Countries or territories	Number of DALYs [No. (95% UI)]
China	1,126,038 (889,923–1,370,837)	China	25,176,256 (20,271,928–30,148,436)
India	375,303 (293,418–452,341)	India	9,594,818 (7,622,796–11,561,024)
Indonesia	224,573 (185,474–260,053)	Indonesia	5,797,687 (4,814,441–6,741,163)
Russia	178,287 (140,550–221,370)	Russia	3,561,869 (2,928,624–4,191,895)
Bangladesh	84,019 (60,449–108,191)	Bangladesh	1,939,862 (1,420,433–2,429,506)
USA	80,997 (62,112–102,290)	Vietnam	1,882,791 (1,443,503–2,324,277)
Viet Nam	79,219 (61,654–98,261)	Pakistan	1,754,278 (1,419,399–2,198,106)
Brazil	68,354 (57,128–79,571)	USA	1,722,555 (1,392,242–2,046,073)
Japan	63,733 (46,148–83,012)	Brazil	1,599,546 (1,370,322–1,812,722)
Pakistan	62,425 (49,256–78,184)	Japan	1,218,403 (980,743–1,475,615)
ASMR [No. (95% UI)]		ASDR [No. (95% UI)]	
North Macedonia	142.76 (105.37–187.27)	Nauru	3,223.45 (2,461.86–4,130.88)
Mongolia	134.57 (101.96–174.56)	Mongolia	3,111.56 (2,378.13–4,060.13)
Indonesia	128.32 (105.53–149.57)	Vanuatu	3,056.07 (2,281.04–4,064.91)
Nauru	121.65 (93.59–155.29)	Solomon Islands	2,963.74 (2,142.6–3,915.79)
Solomon Islands	119.65 (84.87–157.04)	Kiribati	2,871.87 (2,068.35–3,723.12)
Montenegro	119.23 (89.01–149.68)	Indonesia	2,703.47 (2,253.29–3,118.38)
Vanuatu	118.9 (86.73–161.98)	Mozambique	2,449.83 (1,862.13–3,125.45)
Myanmar	111.6 (87.2–136)	North Macedonia	2,389.32 (1,818.16–3,034.2)
Kazakhstan	105.91 (85.4–127.57)	Myanmar	2,369.47 (1,869.94–2,867.25)
Kiribati	103.52 (74.77–132.74)	Madagascar	2,268.19 (1,591.31–2,972.99)
ASMR-related EAPC [No. (95% UI)]		ASDR-related EAPC [No. (95% UI)]	
North Macedonia	142.76 (105.37–187.27)	Nauru	3,223.45 (2,461.86–4,130.88)
Mongolia	134.57 (101.96–174.56)	Mongolia	3,111.56 (2,378.13–4,060.13)
Indonesia	128.32 (105.53–149.57)	Vanuatu	3,056.07 (2,281.04–4,064.91)
Nauru	121.65 (93.59–155.29)	Solomon Islands	2,963.74 (2,142.6–3,915.79)
Solomon Islands	119.65 (84.87–157.04)	Kiribati	2,871.87 (2,068.35–3,723.12)
Montenegro	119.23 (89.01–149.68)	Indonesia	2,703.47 (2,253.29–3,118.38)
Vanuatu	118.9 (86.73–161.98)	Mozambique	2,449.83 (1,862.13–3,125.45)
Myanmar	111.6 (87.2–136)	North Macedonia	2,389.32 (1,818.16–3,034.2)
Kazakhstan	105.91 (85.4–127.57)	Myanmar	2,369.47 (1,869.94–2,867.25)
Kiribati	103.52 (74.77–132.74)	Madagascar	2,268.19 (1,591.31–2,972.99)

According to the cluster analyses, 32, 110, 59, and 3 countries or territories were grouped into “minor increase” (Indonesia, the Philippines, Omen, Kuwait, etc.), “remained stable” (Togo, Iraq, Cuba, Sudan, etc.), “minor decrease” (Japan, Italy, Brazil, etc.), and “significant decrease” (Singapore, Estonia, Korea, etc.), respectively (Supplementary Table 1).

### The global burden of stroke attributable to HSBP by age and sex

**Figure 4:**
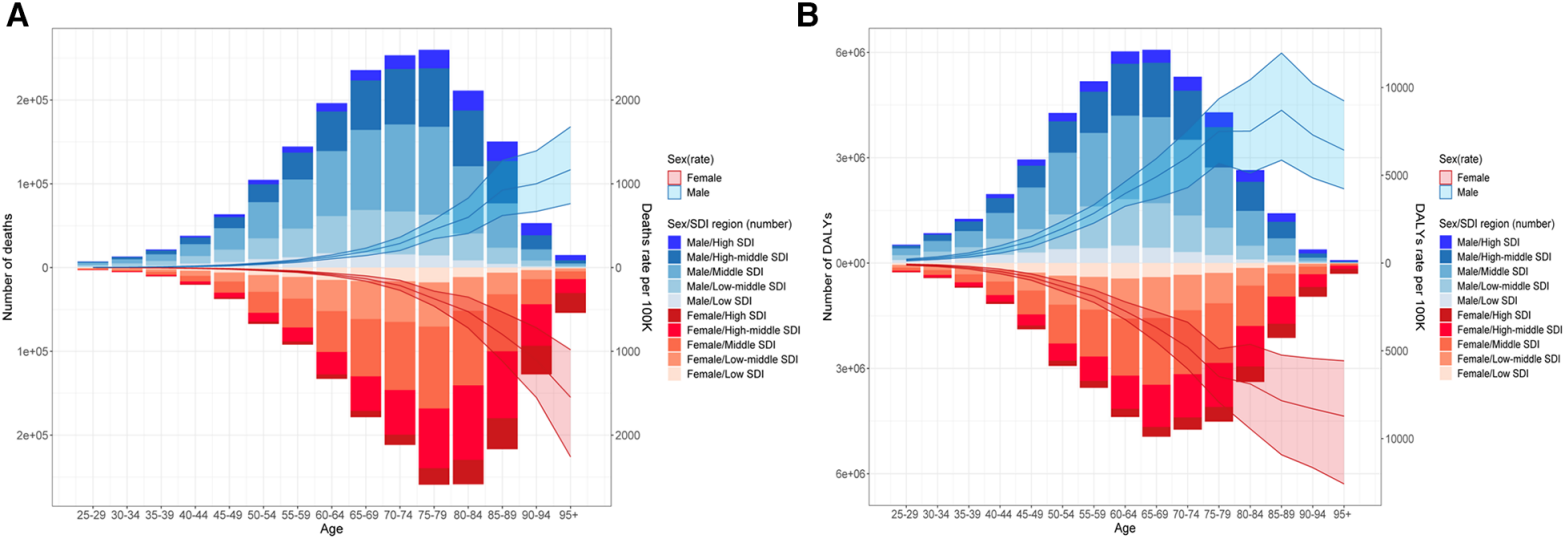
Age distribution of deaths (**A**) and DALYs (**B**) of stroke attributable to HSBP in 2019. The bar was the number of deaths and DALYs. The line with 95% UI represents the mortality rate and DALY rate.

The number of deaths by age experienced an upward trend but decreased since then in both men and women; those aged 70–84 years accounted for most of the deaths, with the peak in those aged 75–79 years. The regions with middle to high-middle SDI had more ASMR. Men and women had a similar ASMR, wherein those aged ≥95 years had an increased ASMR and shared the peak rate (Figure 4A). The number of ASDR had a similar pattern to ASMR, except for the peak point in those aged 65–69 years and for a higher DALY in those aged 60–74 years; ASDR increased in those aged 85–89 years and experienced a decline since then in men aged ≥90 years, while it experienced an upward trend and reached a maximum in women aged ≥95 years (Figure 4B).

**Figure 5:**
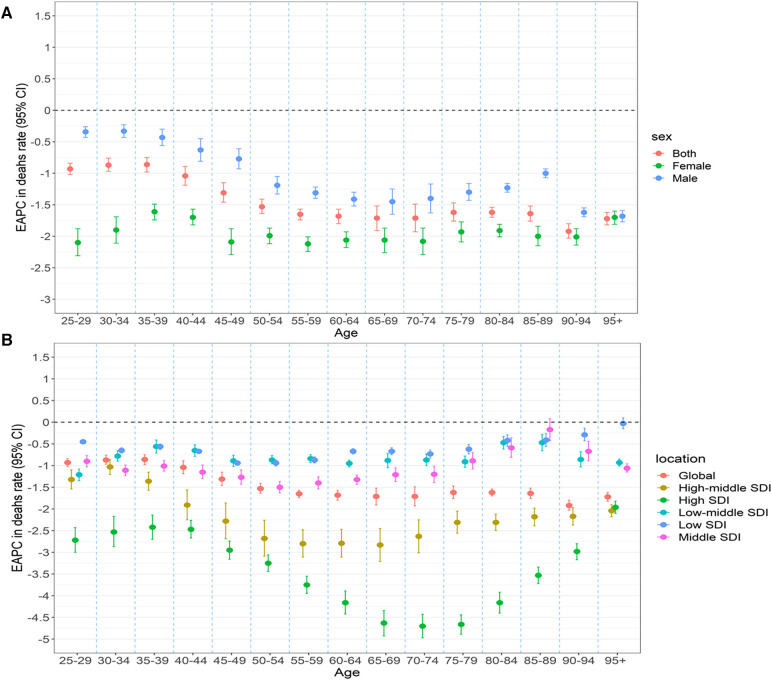
Age distribution of stroke attributable to HSBP trend in mortality rate in 1990–2019 by sex (**A**) and location (**B**) HSBP.

Globally, between 1990 and 2019, ASMR experienced a decline in all age groups, with the most declines in those aged 90–94 years; it also experienced a decline in both women and men (Figure 5A), had a decline across the five SDI regions, with the biggest decline in those aged 70–74 years in high SDI regions (Figure 5B), which was similar to the pattern of ASDR-related EAPCs.

### Factors associated with stoke burden attributable to HSBP

**Figure 6:**
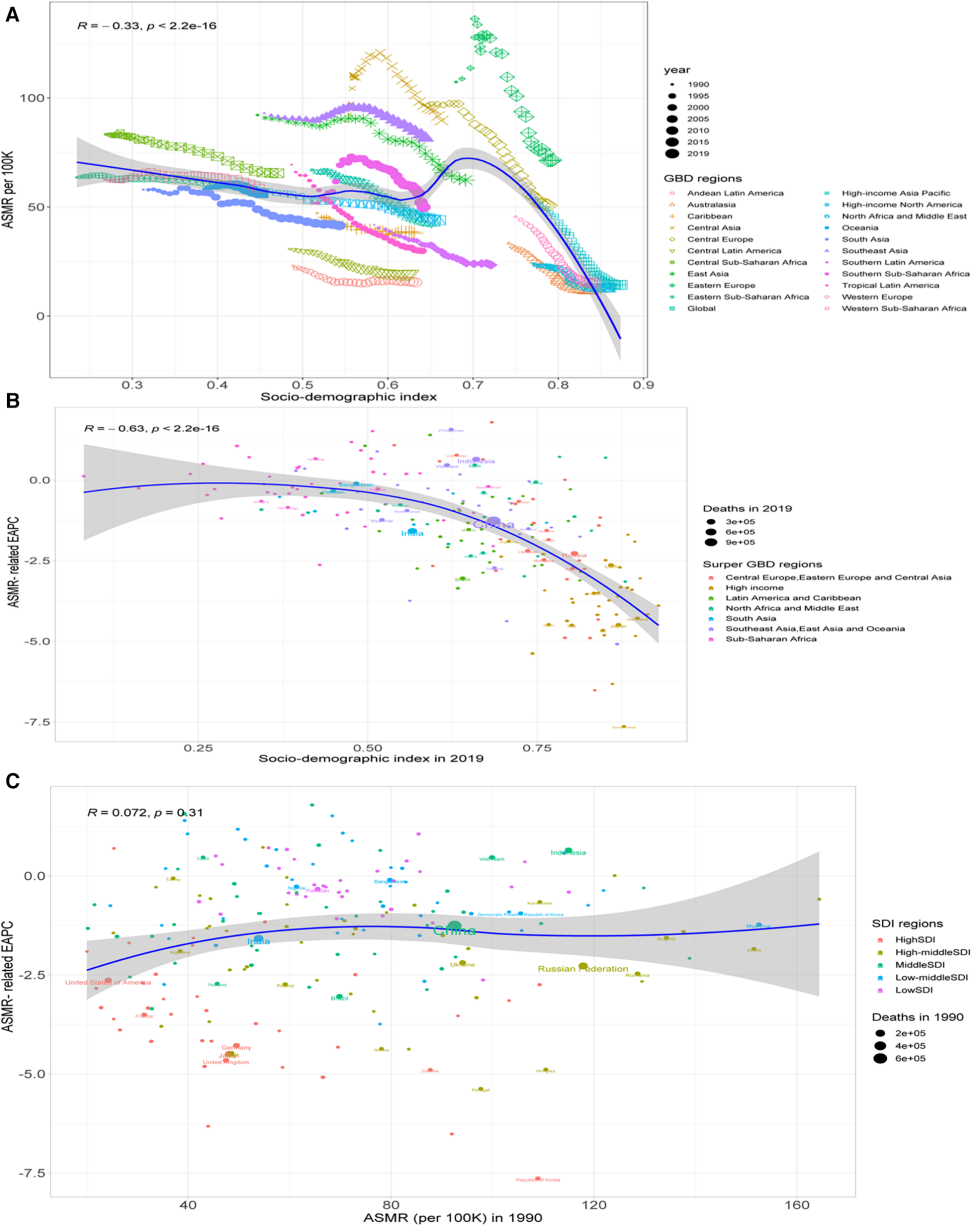
Correlation between the ASMR and SDI (**A**), between the ASMR-related EAPC and the SDI in 2019 (**B**), and between the ASMR-related EAPC and the SDI in 1990 (**C**).

**Figure 7:**
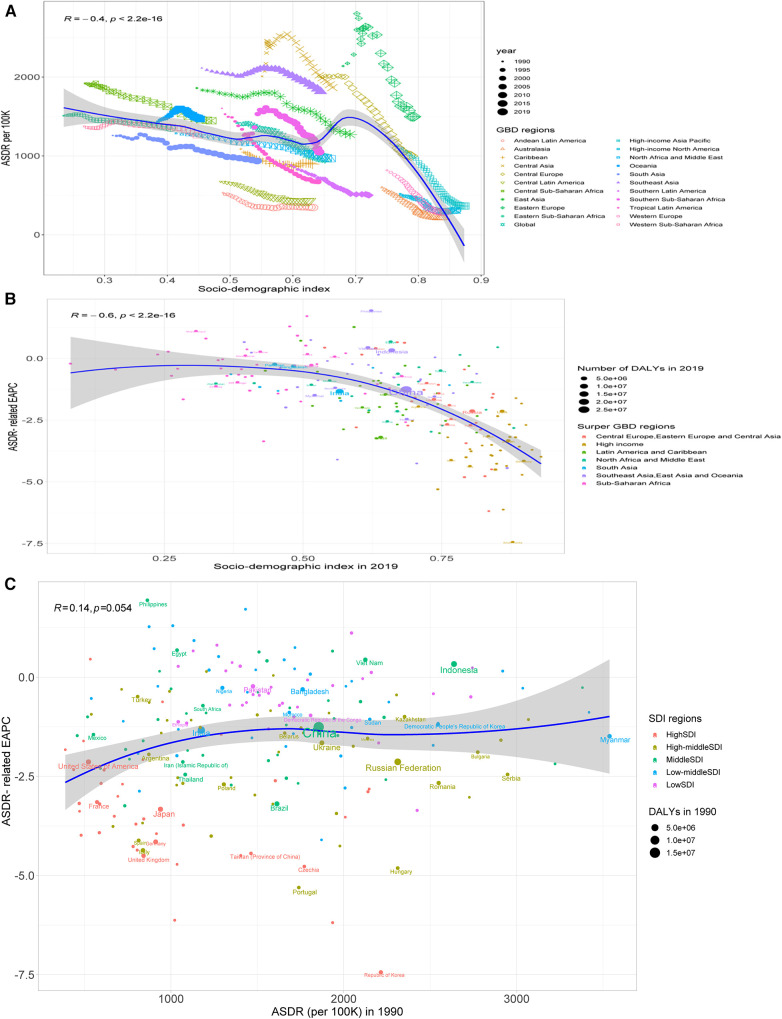
Correlation between the ASDR and SDI (**A**), between the ASDR-related EAPC and the SDI in 2019 (**B**), and between the ASDR-related EAPC and the SDI in 1990 (**C**).

Overall, SDI was negatively related to ASMR (*R* = −0.33, *P *< 0.001), with a sharp decline in SDI >0.7 (Figure 6A); the SDI in 2019 was negatively related to the ASMR-related EAPC (*ρ* = −0.63, *P *< 0.001), particularly the SDI >0.5 (Figure 6B), while the ASMR-related EAPC was not related to the ASMR in 1990 (*R* = 0.072, *P *= 0.31) (Figure 6C). Similar patterns were also observed between SDI and ASDR and the ASDR-related EAPC (Figure 7).

**Figure 8:**
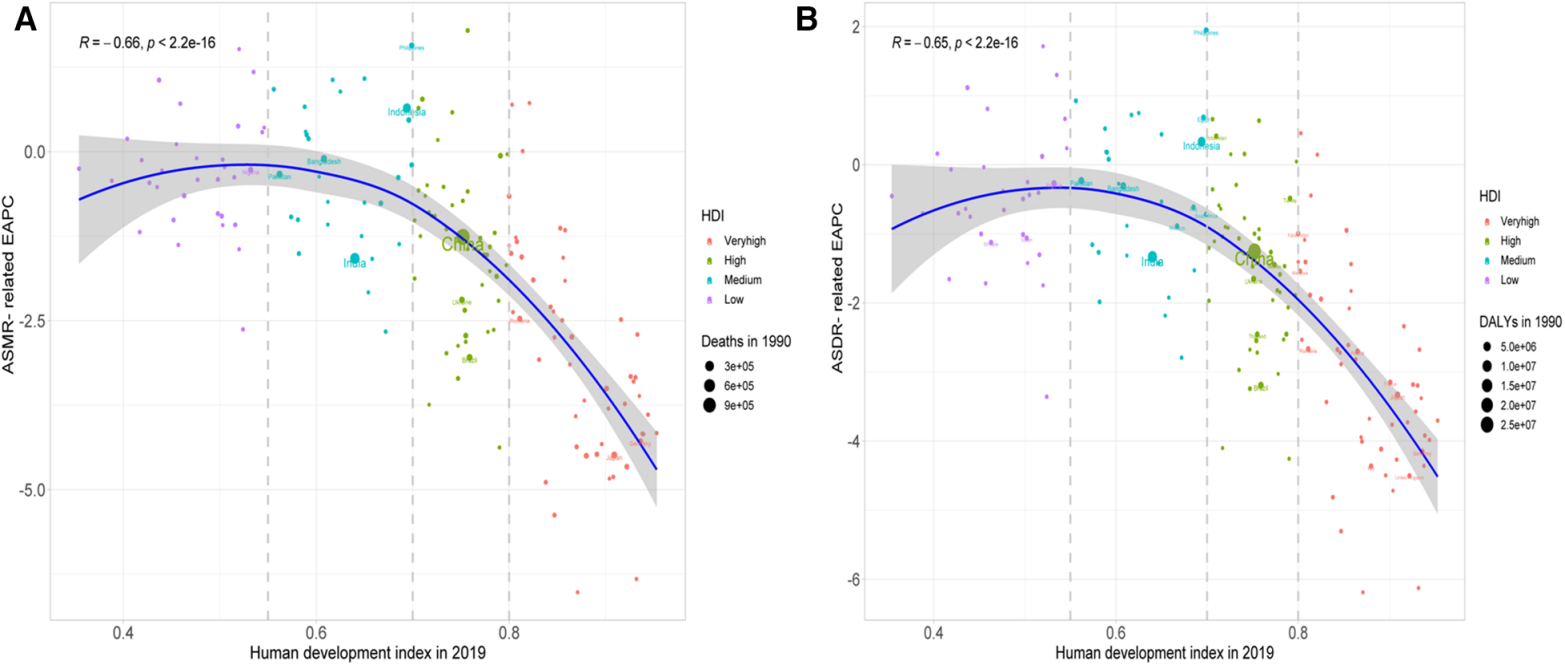
Correlation between the ASMR-related EAPC (**A**) and the ASDR-related EAPC (**B**) and the HDI in 2019.

The HDI in 2019 was negatively related to both ASMR-related EAPC (*ρ* = −0.65, *P *< 0.001) and ASDR-related EAPC (*ρ* = −0.66, *P *< 0.001), particularly the HDI >0.8 (Figure 8).

## Discussion

In the present study, we found that HSBP-related stroke contributed to 52.57% of deaths and 55.54% of DALYs in all strokes; the global deaths and DALYs displayed an upward trend between 1990 and 2019, with a rise of approximately 50% but a decline in both ASMR and ASDR; the percentages of deaths and DALYs accounted for over 40% in all GBD regions in 2019, with the highest percentage level of deaths in Central Asia, the highest percentage of DALYs in Southeast Asia, the highest number of deaths in those aged 75–79 years, and the highest death rates in those aged ≥95 years. SDI was associated with ASMR, the ASMR-related EAPC, and the ASDR-related EAPC in 2019. As far as we know, this is the first time to describe the age, sexual, regional, and national disparities of the global trend in stroke attributable to HSBP, which should provide some comprehensive information for better strategy planning and policymaking to precisely prevent and effectively control stroke attributable to HSBP globally.

A previous study between 1990 and 2016 sheds light on a decline in death rates due to HSBP in most countries, wherein South Korea and Israel recorded the biggest decline; the countries with the highest growth in deaths were China, India, Russia, the United States, and Indonesia; little change showed in the underlying deaths due to HSBP, with over 75% of these deaths responsible for ischemic heart disease and stroke (20). The majority burden in HSBP has switched across regions from high-middle to low SDI. Most of the cause-specific burden of HSBP improved in regions with high SDI but experienced a stagnated downtrend in recent years. Although many causes of death specific to HSBP decreased, cardiovascular disease-related deaths increased in the regions with low, low-middle, and middle SDI (6).

As the most severe risk of stroke, the number and rate of deaths attributable to HSBP theoretically conform to both HSBP and all-caused stroke. SDI is strongly related to health outcomes; HDI is a measurement of summing up mean achievement in human development for a long and healthy life, knowledge, and a decent living standard (http://hdr.undp.org/en/data). High SDI regions have substantially improved the HSBP burden, except for remaining a high burden in other GBD regions (21). Like previous observations on both stroke and HSBP (6, 21), we presented the disproportional associations of SDI and HDI with ASMR/ASDR and the ASMR/DALY-related EAPCs in stroke attributable to HSBP, with a higher proportion of both deaths and DALYs in middle SDI regions accounting for levels over 35% globally and the highest levels of ASMR and DALYs in men in regions with low to middle SDIs. As more than 33% of global deaths and DALYs occur in East Asia and the highest levels of ASMR and ASDR occur in Central Asia, the biggest decline of the ASMR- and ASDR-related EAPCs between 1990 and 2019 correspondingly appeared in high-income Asia Pacific. Such findings here might be illustrated by less awareness and insufficient control of hypertension in low- to middle-income countries (22) and heightened stroke exposure to HSBP in a higher proportion of men (23). In addition, our findings of the size of between-country variations in ASDR and ASMR from stroke attributable to HSBP are consistent with previous observations to stroke (24), which follows that the disparity between several regions may be not because of the HSBP but also disparity in medical technology and hospital capability for stroke treatment.

In our work, the ASMR and ASDR in stroke attributable to HSBP experienced a decline across the five SDI regions, with the biggest decline related to EAPC in high SDI regions, despite a global decline between 1990 and 2019. Although regional and national disparities were observed, with the largest share of the global burden of stroke attributable to HSBP remaining to be borne by the low to middle SDI countries (China, India, Indonesia, and Russia), the proportion of DALYs, due to the GBD-modeled risk factors, was also notably high. Unlike some previous observations (25, 26), we also found that the global decline of ASMR and ASDR in stroke attributable to HSBP corresponded with the rise in mortality rates in China, Brazil, the United States, European countries, and those aged ≥70 years between 1990 and 2019; these national and age disparities might reflect the increased exposure to HSBP and high levels of fasting plasma glucose and body mass index (27), except for a worrisome awareness in those with uncontrolled blood pressure (28). Our findings highlight the potential to considerably lower the stroke burden by thinking highly of exposure to the most severe risk factor and modifying the risk-related profile.

Our systematic estimate of the global trends of stroke attributable to HSBP between 1990 and 2019 should be useful for the global, regional, and national healthcare policies. With the increasing death rates of cardiovascular diseases (29) and the inadequate primary prevention strategies and measures, the population-wide primary prevention strategies for stroke need to be reinforced worldwide (30). According to the World Stroke Organization, all adults are recommended to understand their risk and related factors of stroke and try to use the free Stroke Riskometer app (31–33); therefore, more quality care and action should be proposed to implement culturally suitable and context-appropriate strategies in different countries, particularly in low- and middle-income countries (34–37).

Previous studies have reported the relationship between stroke and HSBP, although the immense disease burden and the global trend of stroke attributable to HSBP have not been explored so far. We based our study on the latest GBD 2019 dataset to estimate the disease's spatiotemporal trend in its deaths, DALYs, and the age-standardized rates at the age, sexual, global, regional, and national levels. Our observations can make better recommendations for the precise prevention and effective control of stroke attributable to HSBP in the GBD-modeled risk regions and populations. Nevertheless, there were some inherent limitations in this study. Data were not free from the lack of overall, original, and well-quality epidemiological information in most countries; therefore, we could not analyze disease burden by stroke subtypes, the decomposition of the changes in population growth and aging, and the effects of direct or indirect factors related to the incorporated HSBP.

## Conclusion

The global burden of stroke attributable to HSBP increased between 1990 and 2019 due to geographical differences and inequities. Proper control of HSBP and further research on HSBP-related stroke are warranted to be better-targeted prevention and treatment efforts over time, particularly for men aged ≥70 years in East, Central, and Southeast Asia, and in the middle to high SDI regions.

## Data Availability

The original contributions presented in the study are included in the article/**Supplementary Material**, further inquiries can be directed to the corresponding author.
