# Supplementary material for: Global burden of stroke attributable to high systolic blood pressure in 204 countries and territories, 1990–2019
Source: Front Cardiovasc Med. 2024 Apr 26;11:1339910. doi: 10.3389/fcvm.2024.1339910 (PMC11084284; doi:10.3389/fcvm.2024.1339910)
Supplement: Supplementary file 1 [file Table1.docx]

| Supplemental tab. 1 The countries and territories were grouped into “minor increase”, “remained stable, “minor decrease, and “significant decrease” using the model of cluster analysis | | | | |
| --- | --- | --- | --- | --- |
| Minor increase | Remained stable | | Significant decrease | Minor decrease |
| Mozambrque | Belize |  | Singapore | New Zealand |
| Cameroon | Bahamas | Republic of Moldova | Estonia | Brunei Darussalam |
| Zimbabwe | Cook lslands | Andorra | Republic of Korea | Denmark |
| Dominican Republic | Sri Lanka | Paraguay |  | Iceland |
| Azerbaijan | China | Argentina |  | Malta |
| Philippines | Lebanon | Grenada |  | Finland |
| Lesotho | Peru | Nicaragua |  | Israel |
| United Republic of Tanzania | Barbados | Bulgaria |  | Japan |
| Indonesia | Malaysia | Cambodia |  | Switzerland |
| Burkina Faso | Georgia | Cuba |  | Cyprus |
| Oman | Mauritania | Qatar |  | Maldives |
| Mongolia | Congo | Ukraine |  | Norway |
| Zambia | Northern Mariana lslands | Syrian Arab Republic |  | Australia |
| Kuwait | Mexico | Guyana |  | Spain |
| Uzbekistan | India | Russian Federation |  | Greece |
| Sao Tome and Principe | San Marino | lran (lslamic Republic of) |  | Mauritius |
| Kenya | Lithuania | Panama |  | Germany |
| Solomon lslands | Comoros | Serbia |  | Slovenia |
| Madagascar | Myanmar | Equatorial Guinea |  | lreland |
| Tonga | Albania | Tokelau |  | Hungary |
| Chad | Namibia | Benin |  | Czechia |
| Eswatini | Burundi | Haiti |  | Luxembourg |
| Ghana | Bolivia (Plurinational State of) | Senegal |  | Taiwan (Province of China) |
| Papua New Guinea | Suriname | Niger |  | United Kingdom |
| Egypt | Saint Vincent and the Grenadines | Central African Republic |  | Italy |
| Viet Nam | Niue | Nigeria |  | Austria |
| Gambia | Democratic Republic of the Congo | Nauru |  | Portugal |
| Timor-Leste | Botswana | United States Virgin lslands |  | Sweden |
| Honduras | Afghanistan | Tuvalu |  | Poland |
| Taiikistan | Sudan | Vanuatu |  | Kyrgyzstan |
| Turkmenistan | Yemen | Pakistan |  | Saint Lucia |
| Guiea | Tunisia | Libya |  | Armenia |
|  | Saudi Arabia | Jamaica |  | Latvia |
|  | Gabon | Liberia |  | El Salvador |
|  | Angola | Dominica |  | Saint Kitts and Nevis |
|  | Uganda | Cote d'lvoire |  | Slovakia |
|  | Bhutan | South Sudan |  | Romania |
|  | Nepal | Mali |  | Uruguay |
|  | Democratic People's Republic of Korea | Malawi |  | Algeria |
|  | Lao People's Democratic Republic | lraq |  | Chile |
|  | North Macedonia | South Africa |  | Bahrain |
|  | Ethiopia | Eritrea |  | Trinidad and Tobago |
|  | Kazakhstan | Morocco |  | Thailand |
|  | Ecuador | Kiribati |  | Puerto Rico |
|  | Bosnia and Herzegovina | American Samoa |  | Costa Rica |
|  | Seychelles | Fiji |  | Belgium |
|  | Antiqua and Barbuda | Samoa |  | United States of America |
|  | Djibouti | Micronesia (Federated States of) |  | Jordan |
|  | Guam | Guatemala |  | Bermuda |
|  | Palestine | Palau |  | Brazil |
|  | Belarus | Guinea-Bissau |  | United Arab Emirates |
|  |  | Somalia |  | Monaco |
|  |  | CaboVerde |  | Greenland |
|  |  | Togo |  | France |
|  |  | Bangladesh |  | Netherlands |
|  |  | Sierra Leone |  | Canada |
|  |  | Marshall lslands |  | Colombia |
|  |  | Turkey |  | Rwanda |
|  |  | Montenegro |  | Croatia |
